# Limited cross-variant neutralization after primary Omicron infection: consideration for a variant-containing booster

**DOI:** 10.1038/s41392-022-01146-0

**Published:** 2022-08-22

**Authors:** Harold Marcotte, Lennart Hammarström, Qiang Pan-Hammarström

**Affiliations:** Department of Biosciences and Nutrition, Karolinska Institutet, 14157 Huddinge, Sweden

**Keywords:** Infectious diseases, Therapeutics

In a recent study published in Nature, Suryawanshi et al. showed that primary Omicron infection only elicits a mild humoral immune response and limited cross-variant neutralization in non-vaccinated individuals.^1^ On the other hand, vaccinated individuals developed high neutralizing antibody titers against all variants of concern (VOCs) following breakthrough Omicron infections. Hence, Omicron infection can enhance existing immunity induced by vaccination but may not prevent reinfection by other VOCs in non-vaccinated individuals.

The Coronavirus disease 2019 (COVID-19) pandemic occurred in successive waves of infection globally, starting with the WA1 strain and followed by the emergence of SARS-CoV-2 VOCs Alpha, Delta, and Omicron. In addition, large regional outbreaks in Southern Africa and South America were caused by VOCs Beta and Gamma, respectively. Mutations in the spike and receptor binding domain (RBD) of VOCs may lead to changes in the structure of the protein and result in stronger binding of the virus to the receptor, increased risk of transmission, as well as a reduction in neutralization susceptibility by antibodies. The emergence of the SARS-CoV-2 Omicron variant has presented a severe threat to the effectiveness of vaccines and therapeutics antibodies.^2,3^ The original Omicron variant (B.1.1.529) contains approximately 37 mutations in the spike glycoprotein, including 15 in RBD. The Omicron variant is constantly evolving and has been divided into five lineages, with BA.2 accounting for the majority of Omicron cases globally and with BA.4 and BA.5 rapidly increasing in several countries in Europe and North America. The Omicron variant is highly transmissible but usually causes milder symptoms compared to other VOCs, leading many to question whether Omicron-induced immunity may provide future cross-protection against other variants and thus help end the pandemic.

To answer this question, Suryawanshi et al. first analyzed infections with SARS-CoV-2 WA1, Delta, and Omicron (BA.1) strains in transgenic mice overexpressing the human angiotensin-converting enzyme 2 (K18-hACE2). Intranasal inoculation of SARS-CoV-2 WA1 and Delta caused significant weight loss and hypothermia in mice. However, symptoms in mice infected with Omicron were mild, including a slight increase in body temperature but no weight loss. During the one-week trial, all Omicron-infected animals survived, but 60% of the Delta- and 100% of the WA1-infected mice reached humane endpoints. Unlike WA1 and Delta, Omicron replicated to low levels in the upper airways and the lungs of infected mice, resulting in a reduction of expression of pro-inflammatory cytokines and T cells activation.

**Fig. 1:**
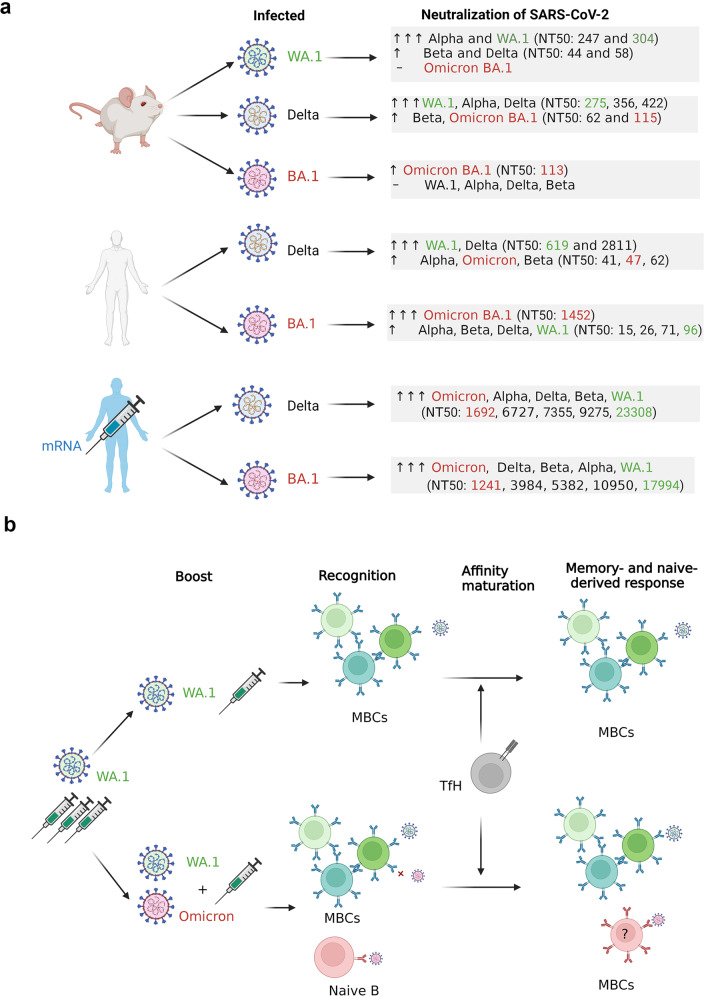
Cross-variant neutralization of SARS-CoV-2 isolates and consideration for boosting the immune response to SARS-CoV-2 variants. **a** Mice were infected with 1 × 10^4^ p.f.u. of WA1, Delta, or Omicron. Unvaccinated or vaccinated individuals were infected with likely Delta or Omicron BA.1. The numbers in parentheses indicate the neutralization efficacy (NT50, 50% neutralization titer) of sera. The triple arrows indicate a higher neutralization activity against the indicated SARS-CoV-2 variants than the single arrow. The dash indicates no neutralization activity. The results suggest that the incorporation of Omicron-based immunogens in future multivalent/heterologous COVID-19 vaccination strategies may provide broader protection against VOCs. **b** Booster vaccination with WA.1 Spike has the potential to recruit previously-formed memory B cells, which undergo further affinity maturation through somatic hypermutations in their immunoglobulin genes. Omicron infection in those vaccinated individuals could reactivate those memory B cells that have sufficient affinity to recognize the variant despite many mutations in the RBD and spike. On the other hand, Omicron and WA.1 boost could, in theory, reactivate those memory B cells against more conserved epitopes as well as activate naive B cells specific for the Omicron spike. As the memory B cells have a pre-activated phenotype, they could respond more rapidly and outcompete naïve B cells, therefore the need to demonstrate new specificities derived from the naïve B cell response for a long period following the boost. The red cross indicates a B cell that do not recognize the Omicron variant but can be stimulated by the WA1 strain. MBCs Memory B cells, TfH T follicular Helper cells. The figure was generated using BioRender (https://biorender.com/)

Sera from mice infected with WA1 effectively neutralized WA1 and Alpha and, to a lower level, Beta and Delta, but not Omicron (Fig. 1a). On the other hand, sera from mice infected with Delta were more effective at neutralizing WA1, Alpha and Delta than Beta and Omicron. Interestingly, infection with Omicron only induced neutralizing activity against Omicron but not other VOCs. Though WA1 and Delta exhibited similar inflammatory and replicative capabilities, their neutralization patterns were distinct, highlighting the role of different S proteins in inducing cross-variant neutralization.

Similarly, sera from unvaccinated individuals who had been infected with Omicron BA.1 effectively neutralized Omicron but showed a 15-fold reduction in neutralizing titers against other VOCs (Fig. 1a). Furthermore, sera from 11 matched, unvaccinated individuals infected with Delta showed the highest neutralization of Delta, followed by WA1 but a decreased level of neutralization of Alpha, Beta, and Omicron. Broader neutralization titers against all VOCs tested were observed in sera from vaccinated individuals who experienced Omicron BA.1 or Delta breakthrough infection, with the highest titers observed against WA1 and the lowest against Omicron.

In summary, the work from Suryawanshi et al. demonstrated that Omicron infection boosts preexisting immunity induced by COVID-19 vaccines but may not confer broad protection against other VOCs in unvaccinated people. The low cross-variant neutralization activity induced by Omicron may be a consequence of its highly mutated S protein or a reduced capacity of replication. The authors thus suggest the inclusion of Omicron- and Delta-based immunogens in future multivalent or heterologous COVID-19 vaccination approaches to provide broader protection against SARS-CoV-2 variants.

Muecksch et al. recently showed that people that received three doses of an mRNA vaccine based on the original (WT) virus have a diverse memory B cell repertoire that can respond rapidly and generate more potent and broader neutralizing antibodies capable of clearing variants, including Omicron BA.1.^4^ The third dose of mRNA vaccines is effective in protecting individuals against severe disease, although the level is insufficient to prevent breakthrough infection in many cases. The plasma of individuals receiving three doses of mRNA vaccines or a combination of inactivated and mRNA vaccines were shown to neutralize BA.1 but with titers 32-fold lower compared to the wild-type strain.^5^ Furthermore, two recent studies showed that sera from individuals who received three doses of vaccines (Pfizer, AstraZeneca, or CoronaVac) and from vaccinated individuals with BA.1 breakthrough infection have a reduced ability to neutralize BA.4, BA.5, and BA.2.12.1 compared with BA.1 and BA.2 due to RBD mutations involving L452R and F486V (BA.4/5) and L452Q (BA.2.12.1).^2,3^ They found that BA.1 Omicron breakthrough infections mainly reactivate WT-induced memory B cells, reducing the diversity of antibodies, and possibly facilitating the emergence of new mutants.^2^ Omicron may thus evolve to escape the humoral immunity induced by BA.1 infection, suggesting that BA.1-derived vaccine boosters may not protect against new Omicron subvariants. Moderna and Pfizer-BioNTech recently developed a bivalent Omicron-containing booster vaccine that induced a higher neutralizing antibody response against omicron BA.1 compared to mRNA-1273, but threefold lower neutralizing antibody response against BA.4 and BA.5 than against BA.1. Thus, development of bivalent, or multivalent vaccines that are designed to target the original coronavirus strain, as well as the new Omicron subvariants (BA.4 and BA.5) is desired and such vaccines will hopefully be available for an anticipated autumn/winter booster campaign later this year. Theoretically, a multivalent vaccine would have the capacity to generate immune memory to both conserved and new epitopes (Fig. 1b). However, as the pre-activated memory B cells could respond more rapidly and outcompete naïve B cells, more data will be needed, especially long after infection or vaccination, to demonstrate new specificities derived from the naïve B cell response.
